# Activation of the Nrf2/ARE signaling pathway by probucol contributes to inhibiting inflammation and neuronal apoptosis after spinal cord injury

**DOI:** 10.18632/oncotarget.19107

**Published:** 2017-07-08

**Authors:** Zipeng Zhou, Chang Liu, Shurui Chen, Haosen Zhao, Kang Zhou, Wei Wang, Yajiang Yuan, Zhuo Li, Yue Guo, Zhaoliang Shen, Xifan Mei

**Affiliations:** ^1^ Department of Orthopedics, First Affiliated Hospital of Jinzhou Medical University, Jinzhou, China; ^2^ Department of Endocrinology, First Affiliated Hospital of Jinzhou Medical University, Jinzhou, China; ^3^ Department of Orthopedics, Second Hospital of Jinzhou, Jinzhou, China

**Keywords:** inflammation, spinal cord injury, Nrf2/ARE, apoptosis, probucol, Autophagy

## Abstract

The nuclear erythroid 2-related factor 2 (Nrf2)/antioxidant response element (ARE) signaling pathway plays an essential role in the cellular antioxidant and anti-inflammatory responses. Spinal cord injury (SCI) results in a massive release of inflammatory factors and free radicals, which seriously compromise nerve recovery and axon regeneration. In this study, we examined the efficacy of probucol on anti-inflammatory responses and functional recovery after SCI by activating the Nrf2/ARE signaling pathway. We also investigated the mechanism by which inflammation is inhibited in this process. We found that treatment of injured rats with probucol significantly increased levels of Nrf2, heme oxygenase-1 (HO-1) and NAD(P)H:quinone oxidoreductase-1 (NQO1), while levels of inflammatory cytokines, interleukin-1β (IL-1β), interleukin-6 (IL-6) and tumor necrosis factor-α (TNF-α) were decreased. This was associated with a reduction in neural cell apoptosis and promotion of nerve function recovery. These results demonstrate that the neuroprotective effects of probucol after SCI are mediated by activation of the Nrf2/ARE signaling pathway. These findings indicate that the anti-inflammatory effects of probucol represent a viable treatment for improving functional recovery following SCI.

## INTRODUCTION

Spinal cord injury (SCI) involves two pathophysiological stages consisting of the primary and secondary injuries. The secondary injury is the cause of a series of detrimental effects, including mitochondrial dysfunction, inflammation and oxidative stress, which contribute to neuronal apoptosis and inhibit nerve recovery and axon regeneration [1, 2]. The inflammatory response is a crucial intracellular catabolic process in the secondary injury [3–5]. SCI induces astrocyte proliferation and migration as well as reactive astrocytosis and hypertrophy [6, 7]. Astrocytes also produce a variety of chemokines and cytokines, which participate in immune and inflammatory responses, which lead either directly or indirectly, to neuronal death [8]. Furthermore, glial scar formation hinders axon regeneration [9, 10]. Reduction in the early inflammatory response after SCI has a neuroprotective effect and promotes functional recovery. Therefore, effective prevention of secondary damage by reduction of inflammatory cytokine production and neuronal apoptosis to promote functional recovery after SCI is a key therapeutic approach for improved prognosis.

The nuclear erythroid 2-related factor 2 (Nrf2) molecule binds to the antioxidant response element (ARE), thereby regulating the expression of a large battery of cytoprotective genes involved in the cellular antioxidant and anti-inflammatory responses [11]. Under normal conditions, Nrf2 is sequestered in the cytoplasm by the Kelch-like ECH-associated protein 1 (Keap1) [12]. Following activation, Keap 1 releases Nrf2 and translocates into the nucleus where it activates an antioxidant response element [13, 14]. The Nrf2/ARE signaling pathway regulates more than 200 endogenous protective genes involved in the cellular antioxidant and anti-inflammatory defense systems [11, 15], including the phase II antioxidant/detoxifying enzyme genes and anti-inflammatory co-stimulating and molecular chaperone genes [16]. These protective genes play an important role in improving organizational antioxidant ability as well as exerting anti-toxin, anti-tumor, anti-inflammatory and anti-apoptotic effects [16, 17]. Therefore, activating the Nrf2/ARE signaling pathway after SCI is implicated as a viable strategy for the reduction of inflammation and promotion of neurological function recovery.

Probucol is a bisphenol compound that functions as a lipid-lowering drug with anti-inflammatory and antioxidant properties and is widely used to lower cholesterol and reduce atherosclerosis in the clinic [18, 19]. Furthermore, more and more studies indicate that probucol has neuroprotective effects in some experimental animal models [20]. Many researchers have reported that probucol provides some degree of neuroprotective effects on Alzheimer's disease, Parkinson's disease and other neurodegenerative diseases [21, 22]. Probucol with pleiotropic properties demonstrated potential in ameliorating cerebrovascular dysfunction by modulating inflammatory pathways, redox homeostasis or direct effects on endothelium to modulate blood-brain barrier integrity [23]. Our previous research has demonstrated that probucol reduces neural cell apoptosis by inducing autophagy via inhibition of the mTOR signaling pathway after SCI [24]. Furthermore, the anti-inflammatory effects of probucol are extremely important and studies have confirmed that probucol increases glutathione (GSH) levels via upregulation of glutamate cysteine ligase (GCL) activity, showing the protective effect of mitochondrial dysfunction on oxidative stress [20]. However, few studies have reported the anti-inflammatory effect of probucol mediated via activation of the Nrf2/ARE signaling pathway after SCI.

In this study, we examined the anti-inflammatory, anti-apoptotic, and neuroprotective effects of probucol and functional recovery following SCI through the activation of Nrf2/ARE signaling pathway both *in vivo* and *in vitro*.

We found that probucol reduced apoptosis and decreased the rate of neuron loss by reducing the inflammatory response in a rat model of acute SCI. Furthermore, our results indicated that this effect is mediated through activation of the Nrf2/ARE signaling pathway. This study provides evidence that probucol may be an effective and feasible therapeutic agent for SCI in clinical applications.

## RESULTS

### Probucol increases neuronal survival, decreases the damaged area and promotes functional recovery after SCI

**Figure 1:**
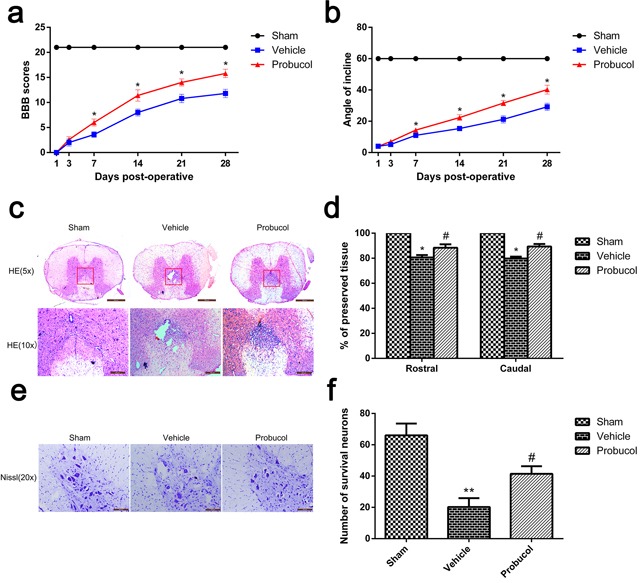
Probucol decreases the damage of tissue structure and the loss of neurons and improves functional recovery after spinal cord injury **a**. The Basso, Beattie and Bresnahan (BBB) scores, *P < 0.05 *versus* the vehicle group. **b**. The inclined plane test scores, *P < 0.05 *versus* the vehicle group, *n* = 5. **c**. HE staining at 7 days. Scale bars are 500 μm (5×) and 200 μm (10×). **d**. Graphic presentation of the percent of preserved tissue in relation to the transverse area of the spinal cord on the seventh postoperative day; columns represent mean ± SD, *P < 0.05 *versus* the sham group, *n* = 5; #*P* < 0.05 *versus* the vehicle group, *n* = 5. **e**. Nissl staining to assess the loss of neurons at 7 days. Scale bars are 100 μm (20×). **f**. Counting analysis of VMN at rostral 5 mm, caudal 5 mm, and lesion site; columns represent mean ± SD, *P < 0.05 *versus* the sham group, *n* = 5; #*P* < 0.05 *versus* the vehicle group, *n* = 5.

The therapeutic effect of probucol on locomotor recovery after SCI was evaluated by assignment of BBB scores and conducting inclined plane tests. Normal scores were assigned in the sham group and there was no significant change in BBB scores in the vehicle and probucol groups at 1 day and 3 days after SCI (Figure 1a). However, the BBB scores were increased in the probucol group at 7 days after SCI. Similarly, we found that the inclined plane test scores in probucol group increased with time at 7, 14, 21, and 28 days after SCI (Figure 1b). These results demonstrate that probucol may influence locomotor functional improvement after SCI. Furthermore, HE staining showed histomorphological differences between the sham, vehicle, and probucol groups at 7 days after SCI (Figure 1c and 1d). Compared with the sham group, there was evident damage to the dorsal white matter and central grey matter in the injury groups. Compared with the vehicle group, the damaged area was decreased in the probucol group which revealed significant therapeutic effects. The effect of probucol on the numbers of motor neurons in the spinal cord was investigated by Nissl staining at 7 days after SCI, and the average number of neurons per section in the thoracic spinal cord of each rat was calculated. Compared with the vehicle group, there was a significantly higher number of spinal front corner motor neurons in the probucol group (Figure 1e and 1f). These results revealed that probucol has a significant neuroprotective effect on motor neurons and enhances tissue preservation following SCI.

### Probucol inhibits astrocytes activation and protects neurons

**Figure 2:**
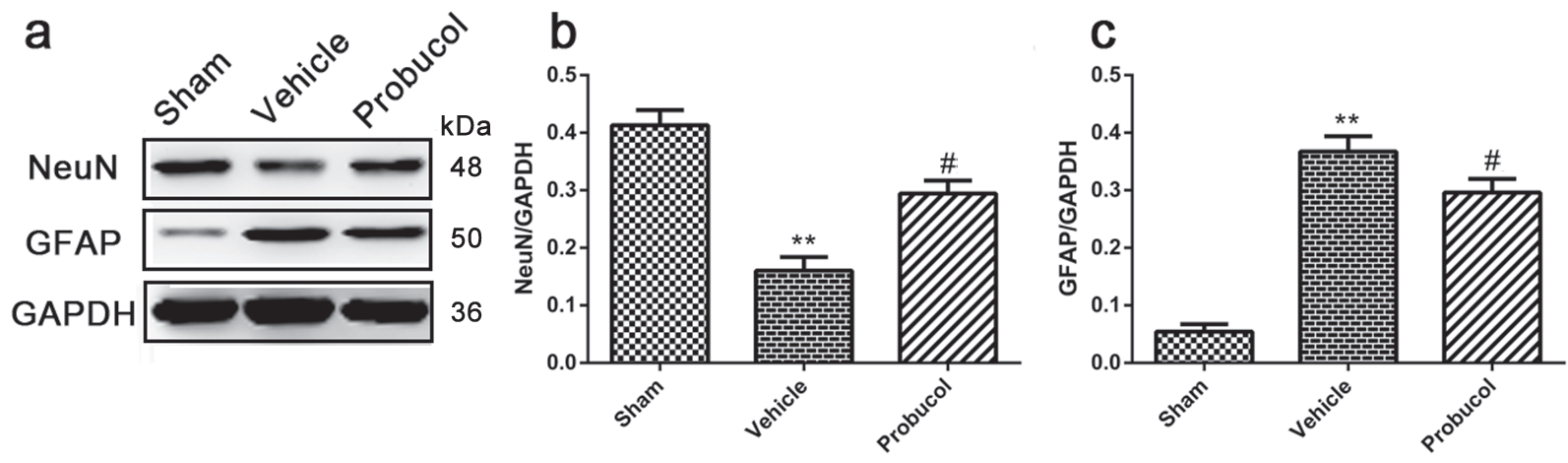
Probucol inhibits astrocytes activation and protects neurons after SCI in rat **a**. **b**. **c**. Representative western blots and quantification data of NeuN, GFAP and GAPDH in each group rats on the seventh postoperative day; columns represent mean ± SD, **P < 0.01 *versus* the sham group, *n* = 5; #*P* < 0.05 *versus* the vehicle group, *n* = 5.

Western blot analysis showed that the expression levels of GFAP (an astrocyte bio-marker) were significantly decreased in the probucol group compared with the vehicle group, while the expression levels of NeuN (a neuron bio-marker) were significantly increased (Figure 2a-2c). These data confirmed that probucol inhibits astrocyte activation and protects neurons in SCI rats.

### Probucol activated the Akt/Nrf2/ARE signaling pathways after SCI

**Figure 3:**
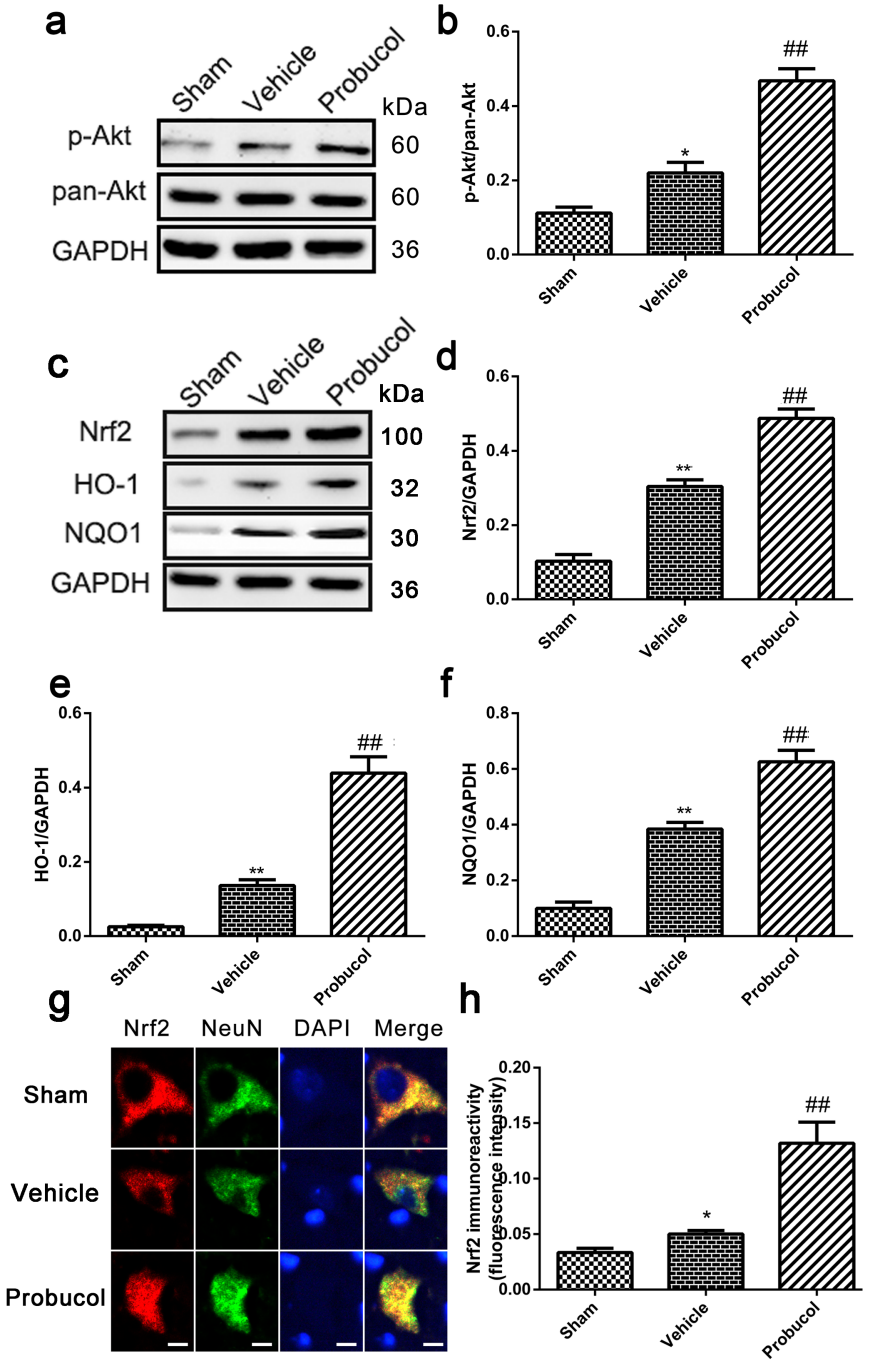
Probucol activated the Akt/Nrf2/ARE signaling pathways after SCI in rat **a**. **b**. Representative western blots and quantification data of p-Akt, pan-Akt and GAPDH in each group rats on the seventh postoperative day; columns represent mean ± SD, *P < 0.05 *versus* the sham group, *n* = 5; ##*P* < 0.01 *versus* the vehicle group, *n* = 5. **c**. **d**. **e**. **f**. Representative western blots and quantification data of Nrf2, HO-1, NQO1 and GAPDH in each group rats on the seventh postoperative day; columns represent mean ± SD, **P < 0.01 *versus* the sham group, *n* = 5; #*P* < 0.05 *versus* the vehicle group, ##*P* < 0.01 *versus* the vehicle group, *n* = 5 **g**. **h**. immunofluorescence analysis was used to detect the staining intensity of Nrf2 at 7 days. Scale bars are 5 μm. *P < 0.05 *versus* the sham group, *n* = 5; ##*P* < 0.01 *versus* the vehicle group, *n* = 5.

Numerous reports have indicated that Nrf2/ARE signaling pathways are essential for the antioxidant and anti-inflammatory properties of probucol [20]. Nrf2 is positively regulated by PI3K/Akt and results in the inhibition of inflammation. To elucidate the mechanism underlying the therapeutic effects of probucol after SCI, we investigated the involvement of the Akt/Nrf2/ARE signaling pathway. The levels of p-Akt, pan-Akt, Nrf2, HO-1, and NQO1 were analyzed by Western blotting (Figure 3a and 3c). The higher expression of the strong antioxidants, HO-1 and NQO1, were induced by activated Nrf2. Expression of p-Akt/pan-Akt, Nrf2, HO-1, and NQO1 was significantly higher after SCI compared with the levels detected in the sham group, thus implying the activation of the Akt/Nrf2/ARE signaling pathway. Compared to the vehicle group, probucol treatment resulted in a remarkably higher ratio of p-Akt/pan-Akt, Nrf2, HO-1, and NQO1 (Figure 3b, 3d-3f). In addition, immunofluorescence analysis showed the expression of Nrf2 in the probucol group was evidently increased (Figure 3g and 3h). These results suggested that probucol activates the Akt/Nrf2/ARE signaling pathway to promote antioxidant and anti-inflammatory responses in acute SCI.

### Probucol reduces inflammation after SCI

**Figure 4:**
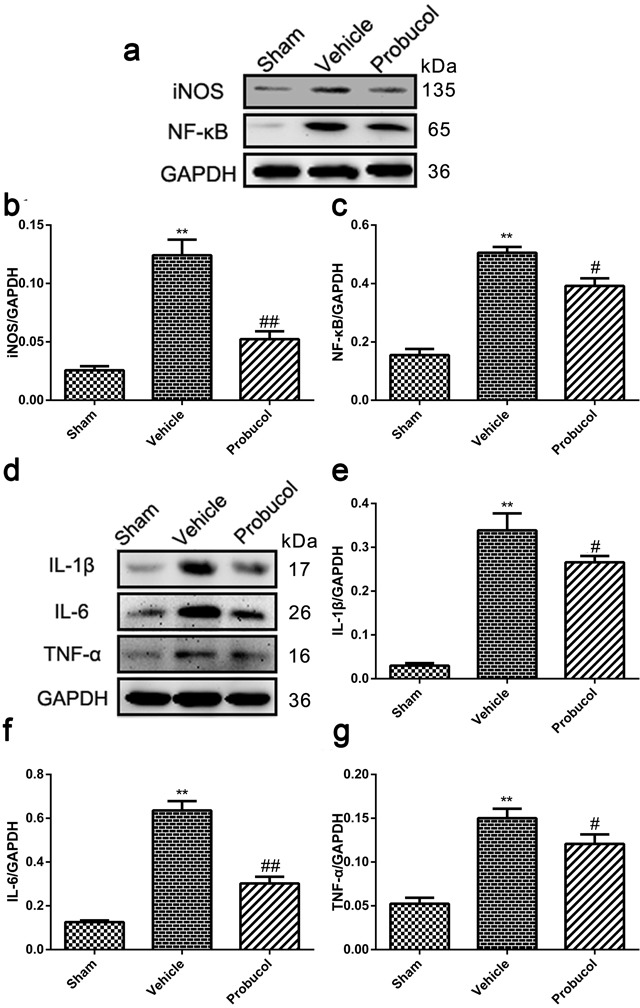
Probucol reduced the inflammation after SCI in rat **a**. **b**. **c**. Representative western blots and quantification data of iNOS, NF-κB and GAPDH in each group rats on the seventh postoperative day; columns represent mean ± SD, **P < 0.01 *versus* the sham group, *n* = 5; #*P* < 0.05 *versus* the vehicle group, ##*P* < 0.01 *versus* the vehicle group, *n* = 5. **d**. **e**. **f**. **g**. Representative western blots and quantification data of IL-1β, IL-6, TNF-α and GAPDH in each group rats on the seventh postoperative day; columns represent mean ± SD, **P < 0.01 *versus* the sham group, *n* = 5; #*P* < 0.05 *versus* the vehicle group, *n* = 5.

Expression of iNOS, NF-κB, IL-1β, IL-6 and TNF-α was analyzed by Western blot to evaluate the potential of probucol to inhibit cellular inflammation (Figure 4a and 4d). Expression of these inflammatory proteins was significantly decreased by probucol treatment after SCI (Figure 4b and 4c, 4e-4g). These results indicated that probucol is an effective therapeutic strategy that inhibits inflammation after acute SCI.

### Probucol attenuates apoptosis caused by acute SCI

**Figure 5:**
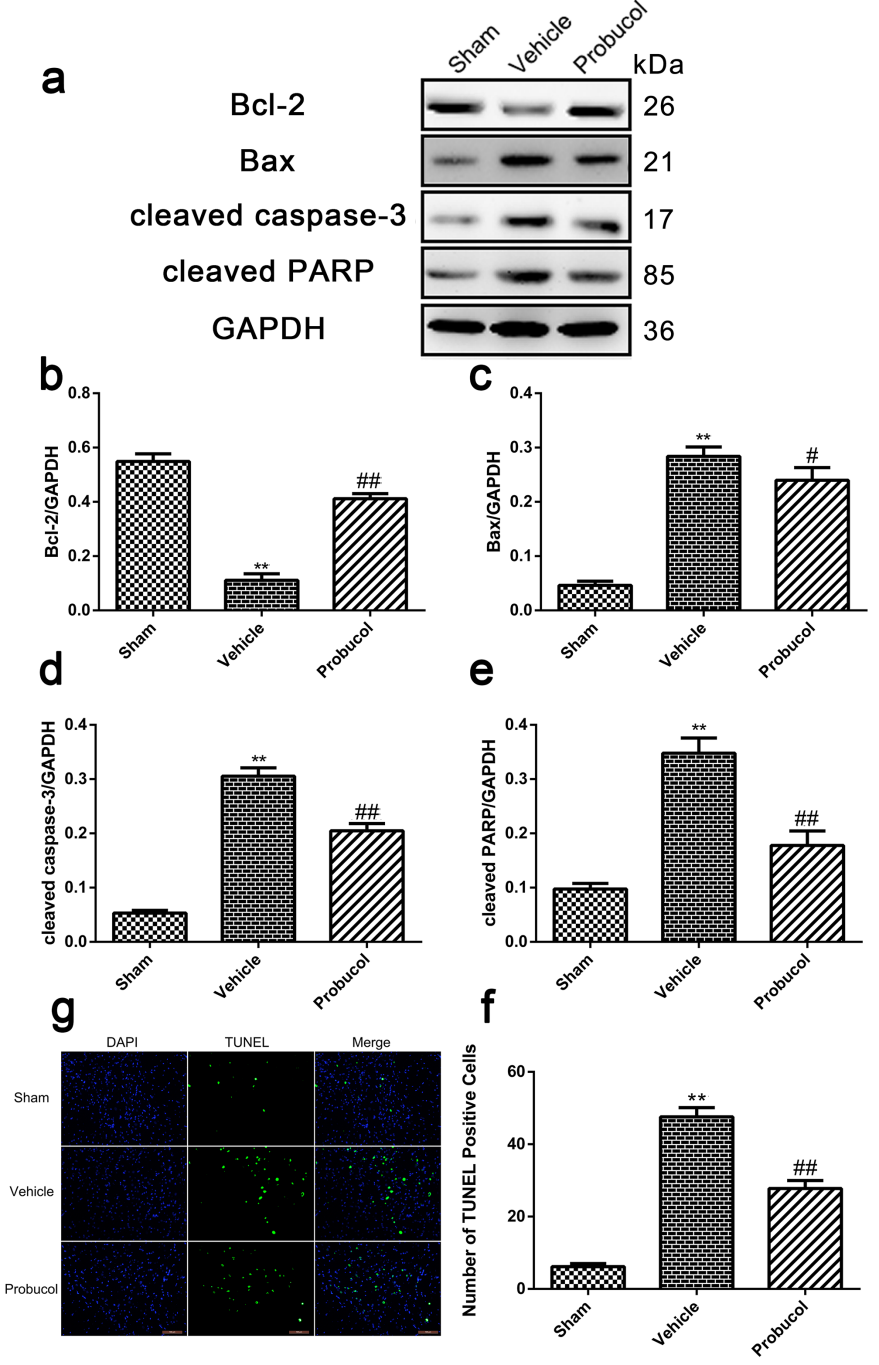
Probucol attenuates apoptosis caused by spinal cord injury in rat **a**. **b**. **c**. **d**. **e**. Representative western blots and quantification data of Bcl-2, Bax, cleaved caspase-3, cleaved PARP and GAPDH in each group rats on the seventh postoperative day; columns represent mean ± SD, *P < 0.05 *versus* the sham group, **P < 0.01 *versus* the sham group, *n* = 5; #*P* < 0.05 *versus* the vehicle group, ##*P* < 0.01 *versus* the vehicle group, *n* = 5. **f**. TUNEL staining to assess the apoptosis at 7 days. g Quantitative estimation of apoptotic and TUNEL cells in each group rats on the seventh postoperative day. Columns represent mean ± SD, **P < 0.01 *versus* the sham group, #*P* < 0.05 *versus* the vehicle group, *n* = 5. Scale bars are 100 μm.

Expression levels of cleaved caspase-3, cleaved PARP, Bax, and Bcl-2 were analyzed by Western blotting. Probucol significantly inhibited the expression of cleaved caspase-3 and cleaved PARP caused by SCI (Figure 5a, 5d and 5e). Similarly, the level of anti-apoptotic protein Bcl-2 was upregulated, while the pro-apoptotic protein Bax was downregulated after treatment with probucol (Figure 5a-5c). The ability of probucol to inhibit cellular apoptosis after SCI was evaluated by TUNEL staining (Figure 5f). A greater number of TUNEL-positive cells was detected in the vehicle group than in the sham group, while there were significantly fewer apoptotic cells in the probucol group (Figure 5g). These results demonstrated that probucol administration inhibits apoptosis effectively after acute SCI.

### Probucol inhibits LPS-induced astrocyte activation

**Figure 6:**
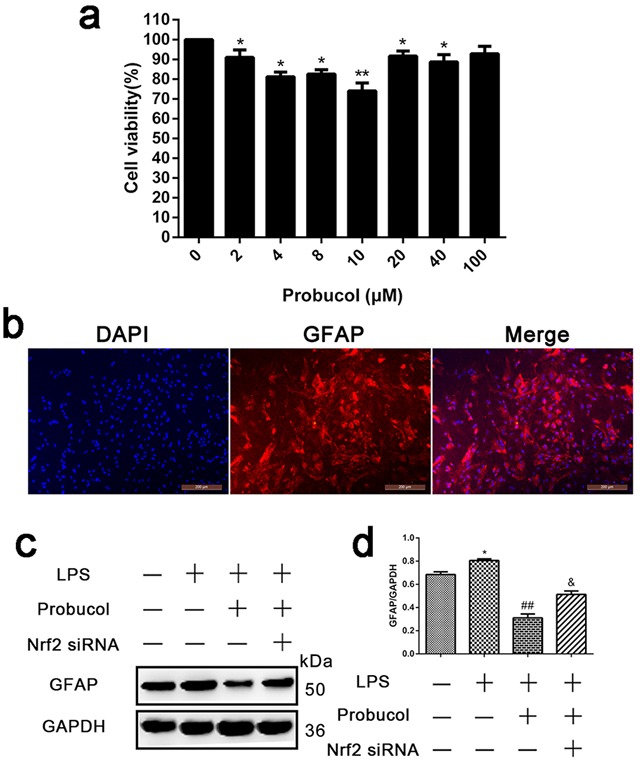
Probucol inhibits LPS-induced astrocytes activation **a**. Representative MTT assay detected primary spinal cord astrocytes viability incubated with probucol (0, 2, 4, 8, 10, 20, 40, 80 and 100 μM) for 24 h. *P < 0.05, **P < 0.01 *versus* the Control group. **b**. Immunofluorescence analysis detected the primary cell purity and the proportion of cells stained with astrocyte-labeled protein GFAP was as high as 90%. **c**. **d**. Representative western blots and quantification data of GFAP and GAPDH in each group of the third passages astrocytes; columns represent mean ± SD, *P < 0.05 *versus* the Control group, *n* = 5; ##*P* < 0.01 *versus* the LPS group, *n* = 5; &*P* < 0.05 *versus* the LPS+Probucol group, *n* = 5.

Primary spinal cord astrocytes were incubated with probucol (0, 2, 4, 8, 10, 20, 40, 80 and 100 μM) for 24 h after lipopolysaccharide (LPS, as an inducer to establish an inflammatory model)-induced for 12h, and cell viability was detected in MTT assays. The result showed that astrocyte viability was significantly reduced after treatment with 10 μM probucol (Figure 6a). The primary cell purity was detected by immunofluorescence analysis. The result showed that the proportion of cells stained with astrocyte-labeled protein GFAP was as high as 90% (Figure 6b). Western blot analysis showed that probucol significantly decreased GFAP expression (Figure 6c and 6d), suggesting that probucol inhibits LPS-induced astrocyte activation. However, Nrf2 siRNA reversed this phenomenon.

### Probucol activates the Nrf2/ARE signaling pathway in LPS-induced astrocytes

**Figure 7:**
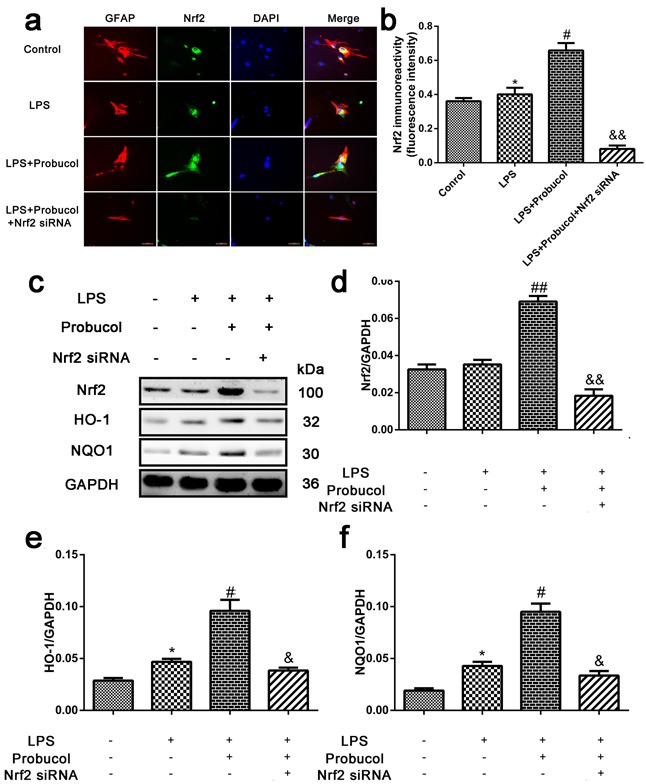
Probucol activated the Nrf2/ARE signaling pathways in astrocytes **a**. **b**. Immunofluorescence analysis was used to detect the staining intensity of GFAP and Nrf2. Scale bars are 50 μm. *P < 0.05 *versus* the Control group, *n* = 5; #*P* < 0.05 *versus* the LPS group, *n* = 5; &&*P* < 0.01 *versus* the LPS+Probucol group, *n* = 5. **c**. **d**. **e**. **f**. Representative western blots and quantification data of Nrf2, HO-1, NQO1 and GAPDH in each group of the third passages astrocytes; columns represent mean ± SD, *P < 0.05 *versus* the Control group, *n* = 5; #*P* < 0.05 *versus* the LPS group, *n* = 5; &*P* < 0.05 *versus* the LPS+Probucol group, *n* = 5.

After SCI, astrocytes produce numerous chemokines and cytokines, which participate in immune and inflammatory responses, causing either direct or indirect neuronal death. Astrocytes were treated with LPS to simulate the inflammatory response after SCI. To confirm that probucol activated the Nrf2/ARE signaling pathway *in vitro*, astrocytes were treated with probucol and Nrf2 siRNA prior to immunofluorescence analysis of the expression levels of Nrf2 (Figure 7a) and Western blot analysis of the expression levels of Nrf2, HO-1 and NQO1 (Figure 7c). Nrf2 expression in astrocytes was evaluated by immunofluorescence analysis (Figure 7a and 7b). A higher level of Nrf2 expression was observed in probucol and LPS co-treated cells compared to control and LPS-treated alone cells. However, Nrf2 siRNA reversed this phenomenon (Figure 7b). In addition, Nrf2 siRNA significantly suppressed activation of the Nrf2/ARE signaling pathway in cells co-treated with probucol and Nrf2 siRNA, and the expression of Nrf2, HO-1 and NQO1 was enhanced by Nrf2 siRNA, indicating that probucol activates the Nrf2/ARE signaling pathway (Figure 7c-7f). These results suggested that the effects of probucol in astrocytes are mediated by activation of the Nrf2/ARE signaling pathway.

### Probucol reduces inflammation in LPS-stimulated astrocytes

**Figure 8:**
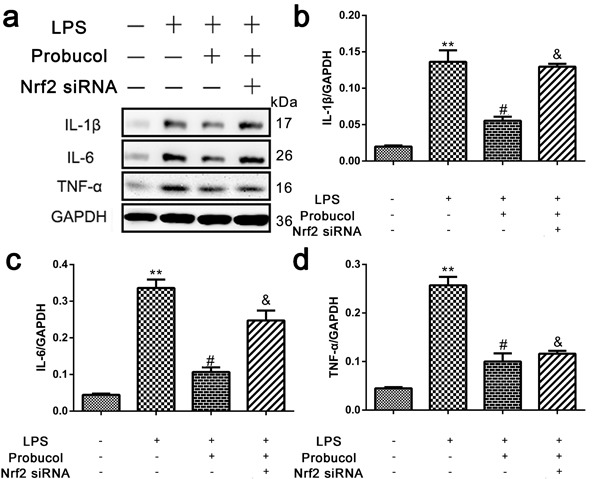
Probucol reduced the inflammation in astrocytes **a**. **b**. **c**. **d**. Representative western blots and quantification data of IL-1β, IL-6, TNF-α and GAPDH in each group of the third passages astrocytes; columns represent mean ± SD, **P < 0.01 *versus* the Control group, *n* = 5; #*P* < 0.05 *versus* the LPS group, *n* = 5; &*P* < 0.05 *versus* the LPS+Probucol group, *n* = 5.

The ability of probucol to inhibit cellular inflammation was confirmed by treating astrocytes with probucol and Nrf2 siRNA prior to Western blot analysis of the expression levels of IL-1β, IL-6 and TNF-α (Figure 8a). Expression of these inflammatory proteins was significantly decreased by probucol treatment after LPS-stimulation of astrocytes and Nrf2 siRNA reversed this phenomenon (Figure 8b-8d). These results indicated that probucol reduces inflammation in astrocytes by activating the Nrf2/ARE signaling pathway.

## DISCUSSION

This study primarily shows that probucol confers neuroprotection against secondary injury in a rat model of SCI. SCI is a major cause of paralysis and death worldwide [25]. Secondary injury is caused by inflammation, oxidative stress and mitochondrial dysfunction, which contribute to neuronal apoptosis and influence nerve recovery and axon regeneration after SCI [1, 2]. The inflammatory response involves the release of a series of inflammatory cytokines, which cause neuronal cell death [4, 5].

Neuronal cell death due to necrosis and apoptosis seriously affects the prognosis of CNS diseases [26]. After acute SCI, the secondary injury especially the inflammatory response results in neuronal apoptosis [27]. Inflammation and oxidation are known to play important roles in apoptosis after SCI. Several studies have indicated that inhibition of neural cell apoptosis plays an essential role in nerve and motor function recovery [28–31]. Apoptosis is an active process of programmed cell death that maintains a stable internal environment [32] via a highly regulated series of gene activation and expression events [33]. Apoptosis is a process of strict polygenic control. The genes involved include the Bcl-2 family, caspase family and cancer genes (C-myc and p53) are highly conserved between species, [34–37]. The development of molecular biology techniques has yielded considerable clarification of the process of cell apoptosis although the precise mechanism remains to be fully elucidated. Furthermore, dysregulated apoptosis may have a direct or indirect relationship with many diseases, such as cancer and autoimmune disorders [38].

In this study, we used probucol to reduce inflammation and inhibit neural cell apoptosis after SCI via activation of Nrf2/ARE signaling pathway. The present study explored the known neuroprotective effects of probucol in the central nervous system. For instance, probucol was found to afford neuroprotection in a 6-OHDA mouse model of Parkinson's disease [22]. In addition, studies have demonstrated that probucol simultaneously inhibits mTORC1 while activating PPAR and AMPK in cellular respiratory chain disease models [39]. Probucol was also reported to reduce neural cell apoptosis by inducing autophagy via inhibition of the mTOR signaling pathway after SCI [24]. Furthermore, it has been demonstrated that succinobucol (a succinyl ester of probucol) increases GSH levels via upregulation of GCL activity (possibly through the activation of the Nrf2/ARE pathway) and is associated with protective effects against mitochondrial dysfunction-derived oxidative stress [20]. This study was designed to explore the hypothesis that the protective effects of probucol after SCI are mediated via the Nrf2/ARE signaling pathway. And many studies suggested that PI3K/AKT signaling pathway involved in the induction of Nrf2/ARE-driven gene expression [40–44]. Our results show that probucol reduces the inflammatory response after SCI by activating AKT and Nrf2/ARE pathway in astrocytes and inhibiting apoptosis leading to improved motor function recovery in rats.

Post-traumatic inflammatory reactions have been shown to contribute to progressive tissue damage after SCI [45]. Following SCI, inflammatory cells in the injured spinal cord tissue produce large amounts of a range of inflammatory mediators. The early stages after SCI are characterized by neutrophil infiltration and expression of cytokines such as TNF-α and IL-1β [46–48]. The interaction between inflammatory cells and mediators may lead to a progressive course of secondary injury, leading to neuronal and glial cell death, axonal destruction and functional loss. Activation of the Nrf2/ARE signaling pathway after SCI regulates genes involved in detoxification and antioxidant defenses [15], which significantly increase expression of the antioxidants HO-1 and NQO1, and reduce expression of inflammatory cytokines TNF-α and IL-1β, resulting in a significant improvement in tissue retention and beneficial role in coordination [17]. Nrf2 translocates from the cytoplasm to the nucleus to promote increased transcription of NQO1 and HO-1, while detoxification of NQO1 quinone and its derivatives also has the effect of coenzyme Q [49]. NQO1 catalytic α-tocopherol quinone generates the powerful antioxidant α-tocopherolhydroquinone. HO-1 is an oxidative stress protein, which causes hemoglobin decomposition to form ferrous iron, carbon monoxide, and biliverdin. Biliverdin and its metabolite bilirubin are physiological free radical scavengers, which resist the damage caused by oxidative stress [50, 51]. Thus, the Nrf2/ARE signaling pathway provides a viable target for enhancing neuroprotective mechanisms following acute SCI.

After SCI, a large number of astrocytes are activated leading to proliferation and produce a range of inflammatory factors, with neuronal cell death initiated at the peak of astrocyte proliferation.

In this study, astrocytes were treated with LPS to simulate the inflammatory response after SCI. LPS-stimulated astrocyte proliferation is associated with activation of the antioxidant protein Nrf2 to combat this inflammatory injury, while promoting increased NQO1 and HO-1 transcription. In our study, probucol significantly increased the expression of Nrf2, NQO1 and HO-1 in damaged astrocytes, while reducing the expression of IL-1β, IL-6 and TNF-α, which play an essential role in the protective effects of Nrf2 against the inflammatory component of SCI. Probucol significantly suppressed the release of inflammatory factors, while Nrf2 was activated and astrocyte activation was reduced. However, Nrf2 siRNA reversed this phenomenon. All of these results indicating that the anti- inflammation effects of probucol are mediated via activating Nrf2.

The importance of reducing inflammation cannot be ignored in the promoting recovery of secondary injury after SCI. Effective reduction in inflammation significantly reduced neuronal apoptosis in rats after SCI, and played a role in the recovery of nerve function and motor function, which effectively improves the prognosis of SCI. *In vitro* investigations showed that probucol reduced astrocyte activation and the release of cytokines after SCI. Meanwhile, *in vivo* investigations showed that probucol activated Nrf2, reduced the release of cytokines and significantly reduced neuronal apoptosis after SCI. Indicating that this treatment reduces inflammation and promotes recovery of nerve function and motor function via activation of the Nrf2/ARE signaling pathway.

In summary, this is the first study to demonstrate that probucol inhibits neuronal apoptosis and improves the functional recovery after SCI via activation of the Nrf2/ARE signaling pathway. Our results suggested that probucol may provide potential therapeutic interventions for reducing inflammatory reaction to attenuate acute SCI.

## MATERIALS AND METHODS

### Spinal cord injury model and drug treatment

Adult female Sprague-Dawley rats (180-220 g) were purchased from Beijing Vital River Laboratory Animal Technology (Beijing, China). All procedures were carried out in accordance with the United States National Institutes of Health Guide for the Care and Use of Laboratory Animal. The animals were maintained under standard temperature conditions (23 ± 0.5°C) with a 12 h light/dark cycle. The modified weight-drop method was used to build an acute SCI rat model as described previously [52]. Briefly, rats were anesthetized with 10% chloral hydrate (0.33 mL/kg). An incision was made along the midline and the spinal cord was exposed by performing a T9/10 laminectomy. The exposed spinal cord was subjected to contusive injury by dropping a 2-mm diameter impactor (10 g) rapidly from a height of 25 mm height. Laminectomy only was performed in the sham group. After SCI, the bladder was massaged three times daily to aid urination until bladder function was recovered and cefazolin sodium (50 mg/kg, i.p.) was administered intramuscularly on three consecutive days. Probucol (Sigma-Aldrich, St. Louis, MO, USA; 2 mL/kg; i.p.) was diluted in 0.9% saline containing 10% dimethyl sulfoxide (DMSO, Sigma-Aldrich) [21] and administered intraperitoneally for seven consecutive days (first administration at 1 h after SCI) in the probucol group. In the vehicle group, the vehicle (0.9% saline with 10% DMSO, i.p.) was intraperitoneally injected at the same time.

### Cell culture

Rat primary astrocytes were extracted from spinal cords of neonatal SD rats (< 48 h). The tissue was isolated under the microscope and minced using fine forceps. The cells were dissociated by incubation with 0.25% trypsin-0.05% EDTA (Gibco Life Technologies, Carlsbad, CA, USA) for 15 min at 37°C. The digestion was terminated by gentle aspiration several times with DMEM (Gibco Life Technologies) medium containing 10% FBS to inactivate the trypsin. Cells were seeded into a 7.5-cm culture dish in DMEM containing 10% FBS (Gibco Life Technologies). After 10 days, oligodendrocytes and microglia were removed by shaking the flask at 200 rpm for 2 h at 37°C. The resultant purified astrocytes were resuspended at 1×10^4^ cells/cm^2^ and added to 96-well plates for cell viability assays, to 75 cm^2^ flasks for Western blot analysis and to 24-well plates for immunocytochemistry experiments. All cells were grown at 37°C under 5% CO_2_. The third passages were used for subsequent experiments.

### RNA transfection and cell treatment

Small interference RNA (siRNA; Qiagen, Cambridge, MA, USA) was utilized to silence the Nrf2 gene. In brief, rat primary astrocytes were transfected with siRNA (30 nM concentration) targeting Nrf2 and non-silencing siRNA using Lipofectamine 2000^TM^ transfection reagent (Invitrogen, Carlsbad). After 6 h, the medium including transfection reagents was decanted. Then, the primary astrocytes were further incubated in the fresh media for 48 h. The Nrf2 low-expressing cells were incubated for subsequent experiments.

### Cell viability assay

The reduction in 3-(4,5-dimethylthiazol-2-yl)-2,5-diphenyltetrazolium bromide to a purple formazan product (MTT) assay was used to determine the cell viability following probucol treatment. Briefly, the cells were treated with probucol (0, 2, 4, 8, 10, 20, 40, 80 and 100 μM) at 37°C after lipopolysaccharide (LPS, 100 ng/mL, Sigma-Aldrich)-stimulation. After 24 h, MTT (20 μL, Sigma-Aldrich) was added to each well and incubated for 4 h at 37°C. Subsequently, 150 μL of DMSO (Sigma-Aldrich) was added to each well and incubated for 10 mins at 37°C. Finally, the absorbance at 490 nm was determined on a Thermo Scientific^TM^ Varioskan^TM^ Flash Multimode Reader (Thermo Fisher Scientific, Inc., Waltham, MA, USA).

### Locomotion tests

At 1, 3, 7, 14, 21, and 28 days post-surgery, locomotion recovery was assessed in rats using the Basso, Beattie, and Bresnahan (BBB) locomotion rating scale which is used to assign scores ranging from 0 points (complete paralysis) to 21 points (normal locomotion) [53]. Each rat was assigned a single score by three highly-trained examiners who were unaware of the group of rats.

The inclined plane test was performed to assess the movement level of the hip, knee, and ankle joints [54]. Briefly, the rats were placed on an inclined plate with a rubber mat to test the ability of rats to maintain posture, grip and the maximum angle at which the rat can hold its position for 5 s without dropping. Measurements were repeated three times for each rat and the highest scores were recorded. Normal rats were able to maintain their position on the inclined plate at a maximum angle of 80 degrees.

### Hematoxylin-eosin (HE) staining and Nissl staining

The spinal cord tissue was removed at seven days post-surgery and perfused with 0.9% NaCl, followed by 4% paraformaldehyde. The spinal cord segments including the site of injury (10 μm transverse frozen sections) were cut using a cryostat microtome (Leica CM3050S, Heidelberg, Germany). Histological changes at the injured site were evaluated following hematoxylin-eosin (HE) staining and the percentage of the preserved area in relation to the total area of each section was calculated [55]. The sections were incubated in 1% cresyl violet (Sigma-Aldrich) at seven days post-surgery for Nissl staining to observe the number of ventral motor neuron (VMN). The Nissl staining positive cells were counted in five randomly selected fields from the area of gray matter in each sample using Image-Pro Plus 6.0 software [56].

### Western blot analysis

The damaged spinal cord (0.5 cm length) at the contusion epicenter was immediately removed at 7 days post-surgery and rinsed three times with 0.1 M phosphate-buffered saline (PBS, pH 7.4). Tissues were homogenized, dissolved in RIPA buffer containing phenylmethane sulfonyl fluoride (PMSF, Beyotime Biotechnology, Nanjing, China) for 20 mins on ice and dissociated using an ultrasonic homogenizer. Tissue homogenates were centrifuged at 12,000 rpm, for 25 min at 4°C. Astrocytes were lysed in RIPA buffer containing PMSF for 10 mins on ice and the protein supernatant was separated by centrifugation at 12,000 rpm for 5 min at 4°C. Approximately 40 μg of total protein was separated by sodium dodecyl sulfate-polyacrylamide gel electrophoresis (SDS-PAGE). Subsequently, the separated proteins were transferred onto a polyvinylidene fluoride (PVDF) membrane and blocked with 5% skim milk powder in TBST (25 mM Tris-HCl, 0.15 M saline, and 1% Tween 20) at room temperature for 2 h. Membranes were then incubated overnight at 4°C with the following primary antibodies: NeuN (1:1,000, Cell Signaling Technology, Danvers, MA, USA), GFAP (1:1,000, Abcam, Cambridge, UK), Nrf2 (1:1,000, Cell Signaling Technology), HO-1 (1:1,000, Abcam), NQO1 (1:1,000, Abcam), pan-Akt (1:1,000, Abcam), p-Akt (1:1,000, Abcam), IL-1β (1:500, Abcam), IL-6 (1:2,000, Abcam), TNF-α (1:1,000, Abcam), Bcl-2 (1:1,000, Cell Signaling Technology), Bax (1:1,000, Cell Signaling Technology), cleaved caspase-3 (1:1,000, Cell Signaling Technology), cleaved PARP (1:1,000, Cell Signaling Technology), iNOS (1:1,000, Cell Signaling Technology), NF-κB (1:1,000, Cell Signaling Technology) and GAPDH (1:1,000, Santa Cruz Biotechnology, Santa Cruz, CA, USA). After washing the membranes three times with TBS (5 min per wash), the membranes were incubated with the appropriate secondary antibodies as follows: HRP AffinPure Goat Anti-Rabbit IgG (1:10,000, EarthOX, San Francisco, CA, USA) and HRP AffinPure Goat Anti-Mouse IgG (1:10,000, EarthOX). Immunoreactive bands were visualized by ChemiDoc-It^TM^TS2 Imager (UVP, LLC, Upland, CA, USA), and quantified by greyscale analysis using by ImageJ2x software.

### Immunofluorescent analysis

Spinal cord sections at seven days post-surgery were incubated with 5% normal goat serum diluted in 0.1% PBS containing Triton X-100 for 2 h at 4°C before being incubated overnight at 4°C with appropriate primary antibodies as follows: anti-NeuN (1:500, Cell Signaling Technology) and anti-Nrf2 (1:100, Cell Signaling Technology). Sections were then washed with 0.1% PBS (3×5 min) at room temperature and incubated with Alexa Fluor^®^568 goat anti-mouse/rabbit IgG (1:400, Life Technology, Carlsbad, California, USA) or Alexa Fluor^®^488 goat anti-mouse/rabbit IgG (1:400, Life Technology) for 2 h at room temperature. Sections were rinsed with 0.1% PBS (3×5 min) and the nuclei were stained with 4, 6-diamidino-2-phenylindole (DAPI, 1:1,000, Abcam) for 10 min. Astrocytes, grown on 24-well plates were washed with ice-cold 0.1% PBS (3×5 min) and fixed with ice-cold 4% paraformaldehyde for 30 min. After washing with ice-cold 0.1% PBS (3×5 min), cells were blocked with 5% normal goat serum diluted in 0.1% PBS containing Triton X-100 for 2 h at 4°C. The cells were then incubated overnight at 4°C with appropriate primary antibodies as follows: anti-Nrf2 (1:100, Cell Signaling Technology) and anti-GFAP (1:200, Abcam). Cells were washed with PBS followed by incubation with Alexa Fluor^®^488 goat anti-mouse/rabbit IgG for 2 h at room temperature. The nuclei were stained with DAPI (1:1,000, Abcam) for 5 min. All slides were observed under a fluorescence microscope (Leica, Heidelberger, Germany). The optical density of fluorescence was analyzed using Image-Pro Plus 6.0 software.

### Terminal deoxynucleotidyl transferase-mediated deoxyuridine triphosphate nick end labeling

DNA fragmentation represents a characteristic of late stage apoptosis. To identify DNA fragmentation in apoptotic cells, transverse frozen sections (thickness, 10 μm) at seven days post-surgery were detected by terminal deoxynucleotidyl transferase-mediated deoxyuridine triphosphate nick end labeling (TUNEL). The apoptotic cells were detected by the In Situ Cell Death Detection Kit (Roche Molecular Biochemicals, Mannheim, Germany) according to the manufacturer's instructions. The proportion of TUNEL-positive neuron cells in three animals per group were analyzed under a fluorescence microscope (Leica, Heidelberger, Germany) [57].

### Statistical analysis

Data are expressed as means ±standard error of the mean (SEM) and analyzed by SPSS 17.0. Statistical significance was examined using Student's t test when there were two experimental groups. When more than two groups were compared, statistical evaluation of the data was performed using one-way analysis of variance (ANOVA) and Dunnett's post hoc test. *P* values < 0.05 were considered to indicate statistical significance.

## Abbreviations

SCI: spinal cord injury
Nrf2: nuclear erythroid 2-related factor 2
ARE: antioxidant response
HO-1: heme oxygenase-1
NQO1: NAD(P)H:quinone oxidoreductase-1
IL-1β: interleukin-1β
IL-6: interleukin-6
TNF-α: tumor necrosis factor-α
Keap1: Kelch-like ECH-associated protein 1
GCL: glutamate cysteine ligase
FBS: fetal bovine serum
DMEM: Dulbecco's modified Eagle's medium
MTT: reduction of 3-(4,5-dimethylthiazol-2-yl)-2,5-diphenyltetrazolium bromide to a purple formazan product
HE: hematoxylin-eosin
VMN: ventral motor neuron
BBB: Basso, Beattie, and Bresnahan
DMSO: dimethyl sulfoxide
PBS: phosphate-buffered saline
PMSF: phenylmethane sulfonyl fluoride
SDS-PAGE: sodium dodecyl sulfate-polyacrylamide gel electrophoresis
PVDF: polyvinylidene fluoride
DAPI: 4, 6-diamidino-2-phenylindole
TUNEL: terminal deoxynucleotidyl transferase-mediated deoxyuridine triphosphate nick end labeling
CNS: central nervous system
